# Optimal diameter reduction ratio of acinar airways in human lungs

**DOI:** 10.1371/journal.pone.0204191

**Published:** 2019-01-31

**Authors:** Keunhwan Park, Yeonsu Jung, Taeho Son, Young-Jae Cho, Noo Li Jeon, Wonjung Kim, Ho-Young Kim

**Affiliations:** 1 Institute of Advanced Machines and Design, Seoul National University, Seoul, Korea; 2 Department of Physics, Technical University of Denmark, Lyngby, Denmark; 3 Department of Mechanical and Aerospace Engineering, Seoul National University, Seoul, Korea; 4 Division of Pulmonary and Critical Care Medicine, Department of Internal Medicine, Seoul National University Bundang Hospital, Seongnam, Korea; 5 Department of Mechanical Engineering, Sogang University, Seoul, Korea; Technion Israel Institute of Technology, ISRAEL

## Abstract

In the airway network of a human lung, the airway diameter gradually decreases through multiple branching. The diameter reduction ratio of the conducting airways that transport gases without gas exchange is 0.79, but this reduction ratio changes to 0.94 in acinar airways beyond transitional bronchioles. While the reduction in the conducting airways was previously rationalized on the basis of Murray’s law, our understanding of the design principle behind the acinar airways has been far from clear. Here we elucidate that the change in gas transfer mode is responsible for the transition in the diameter reduction ratio. The oxygen transfer rate per unit surface area is maximized at the observed geometry of acinar airways, which suggests the minimum cost for the construction and maintenance of the acinar airways. The results revitalize and extend the framework of Murray’s law over an entire human lung.

## Introduction

Fluid transport systems in the form of branching networks have evolved in multicellular organisms to deliver bulk metabolic matter to matter exchange sites [1–7]. In a branching network, a mother branch is divided into numerous terminal daughters. The aggregate cross-sectional area of the vessels of a single generation generally increases with branch generations, and the flow velocity thus decreases. A low flow speed is advantageous for allowing more time for mass transfer at the terminal branches [1]. For instance, the diameter of vascular vessels in the human body decreases from ~1 cm at the aortae to ~10 μm at the capillaries, whereas the aggregate cross-sectional area increases from ~1 cm^2^ to ~10^3^ cm^2^ [1, 8]. However, expanding the cross-sectional area of the daughter vessels can be costly because of the construction and maintenance of redundant channels. Murray's law explains how the costs of running the vascular system can be minimized by controlling the diameter reduction ratio [9, 10]. The same framework has been utilized for rationalizing the observed diameter reduction ratio in the xylem in plants, for which the constructing cost of conduits is the primary factor limiting the expansion of the cross-sectional area of the xylem vessels [11].

**Fig 1:**
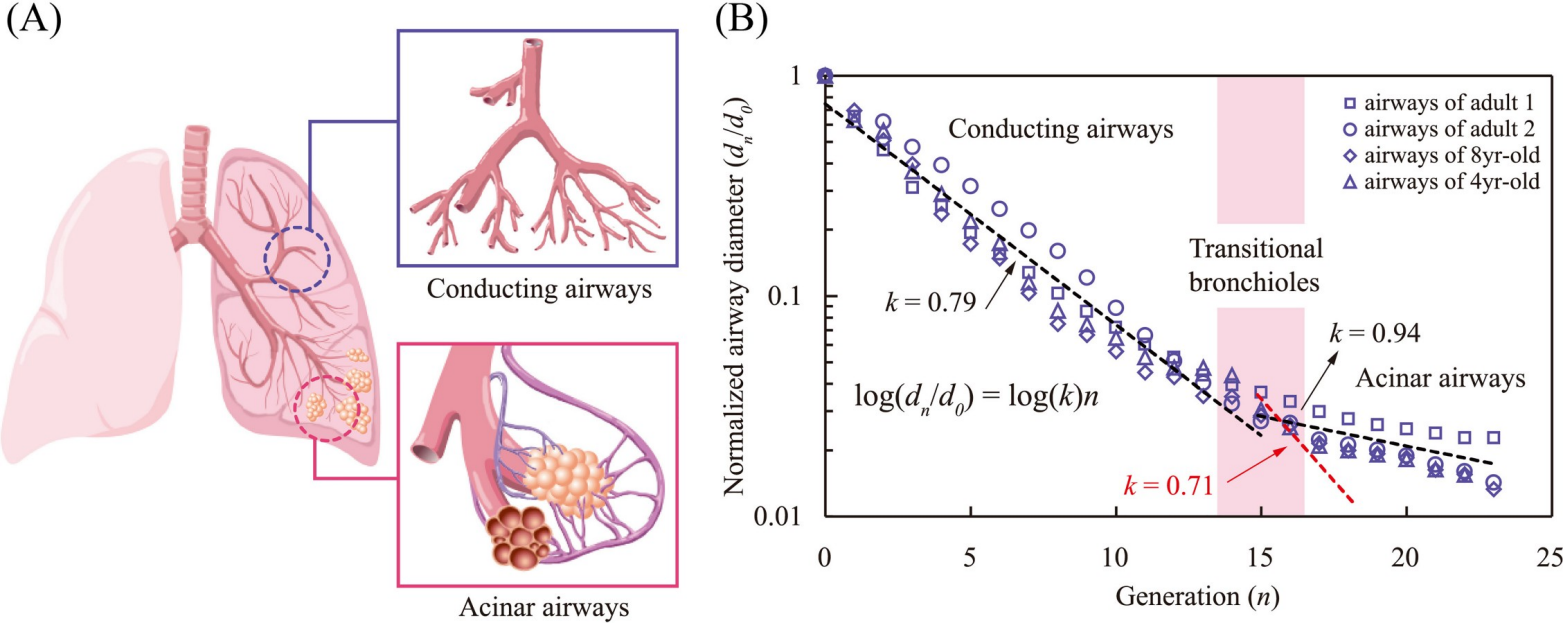
Anatomic schematic of the airways of human lungs. (A) Schematic illustration of conducting airways (blue box) and acinar airways (red box). The hierarchical airway network consists of dichotomous trees with 23 generations. The transferred air diffuses to capillaries enclosed in the alveoli, most of which are attached to the late generations of the airways. The airway lengths of the 16th-23rd generations are 1.33, 1.12, 0.93, 0.83, 0.7, 0.7, 0.7, and 0.7 mm in the order [12], and an average diameter of alveoli is 200 μm [34]. (B) Reduction in normalized airway diameter along with airway generation. The diameter reduction ratio is 0.79 in the conducting airways, whereas it shifts to 0.94 in the acinar airways. Note that Murray’s law for diffusion in insects, *k* = 0.71, cannot explain the acinar airways reduction ratio (red dashed line). Data were taken from Finlay [8] and Weibel [14].

The airway system in a human lung exhibits a similar branching architecture. A single trachea bifurcates into ~2^23^ terminal branches. In this bronchial network, the expansion of the aggregate cross-sectional area of the daughter channels is limited by the airway volume, in such a way that the air transport to the alveoli is maximized for a given amount of inhalation air [12, 13]. Nevertheless, this rationale explains the airway branching only in conducting airways where no gas exchange occurs, as shown in Fig 1.

It has been supposed that the change in gas transfer mode from advection to diffusion is responsible for the transition in the reduction ratio of bronchial airways [10, 14, 15]. Explicit analytical or computational studies, often combined with fractal modeling, focused on the velocity field in airways [16], permeability in acinus [17], breathing irregularity [18], and asymmetric branching [19–22]. The principle of the cost minimization for diffusive mass transfer was developed, which successfully provided the rationale for the spiracle pore networks of insects [23, 24]. However, this model cannot be directly applied to acinar airways because the observed diameter reduction ratio of acinar airways (*k* = 0.94) is much larger than that of Murray's law for diffusion (*k* = 0.71).

Here we present a model for the hitherto unexplained diameter reduction ratio in the acinar airways, *k* = 0.94. With a simplified airway geometry that is amenable to mathematical analysis, our model captures an essential physical picture responsible for the observed diameter reduction ratio.

## Results

We begin with an analysis of the oxygen transfer in human lung airways. During a 2 s period of inhalation, a negative pressure in the pleural cavity induces the expansion of alveoli, and the total volume increase reaches approximately 500 ml [25]. As a result of the increase in the cross-sectional area of the airways, the average speed of the air flow decreases from about 0.5 m/s in the trachea to 1 μm/s in the alveoli. The dominant oxygen transfer mechanism can be examined using the Peclet number, Pe = *ul/D*, the ratio of the advective to the diffusive mass transfer rates, where *l* is the length of a single airway branch (*l* ~ 1 mm), *u* is the speed of the air flow, and *D* is the oxygen diffusion coefficient in air (*D* ~ 0.2 cm^2^/s). Using the data of the cross-sectional area of airways for each generation [8, 14], one can find that Pe < 1 after transitional bronchioles, which suggests a shift in the dominant oxygen transfer mechanism from advection to diffusion [10, 15].

**Fig 2:**
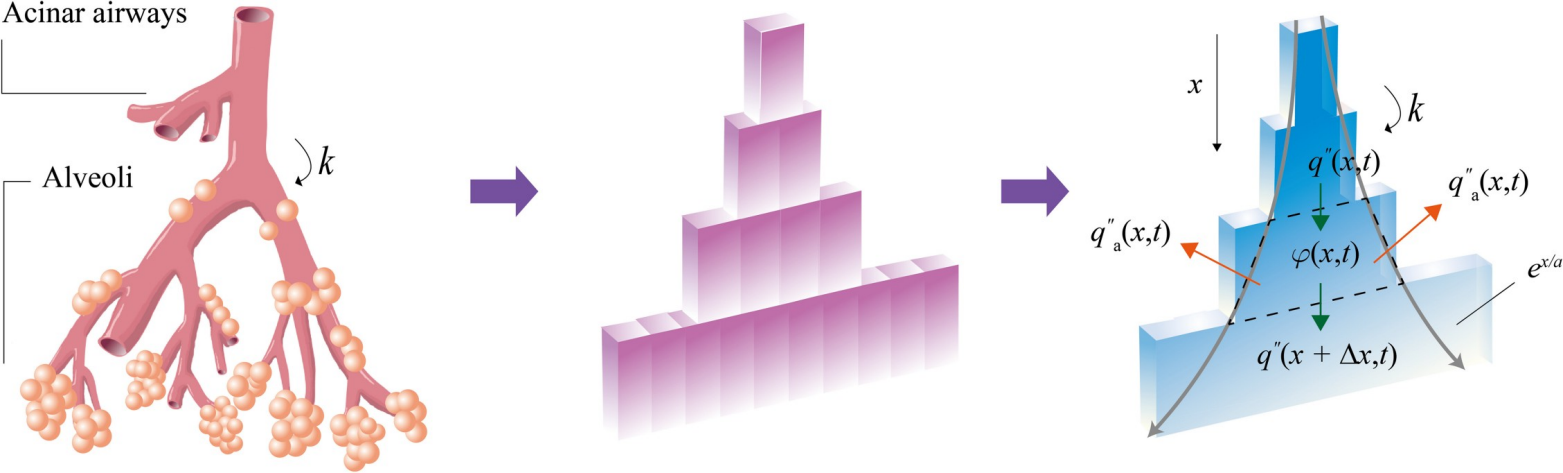
Schematic illustration of a trumpet model for acinar airways. The acinar airways consist of eight generations of airways and alveoli. The acinar airways can be assumed as a bundle of rectangular channels with the identical cross-sectional area in the same generation. The sidewalls of the rectangular channels do not affect the vertical diffusion, so it can be assumed to be a single trumpet channel that expands like an exponential function involving the reduction ratio *k* and single channel length *l*.

We develop a mathematical model of the oxygen transport in the acinar airways. Since the oxygen transfer through the channels via diffusion depends on their cross-sectional area, a trumpet model can be used [26]. We construct a geometric model of the acinar airways as a stepwise channel with a unit depth by arranging all the channels side by side in such a way as to retain an equivalent aggregate cross-sectional area for each generation, as shown in Fig 2. The stepwise channel is further simplified as a diverging duct enclosed by two curved boundaries. Assuming that the transition of diameter reduction ratio occurs near the 16th branch, we can express the cross-sectional area as *A*(*x*) = *A*_16_*e*^*x*/*a*^ with *a* = *l*/ln(2*k*^2^), where *x* is the distance from the 16th branch, *A*_16_ is the total cross-sectional area of the acinar airways at the 16th generation, and *k* is the diameter reduction ratio.

**Fig 3:**
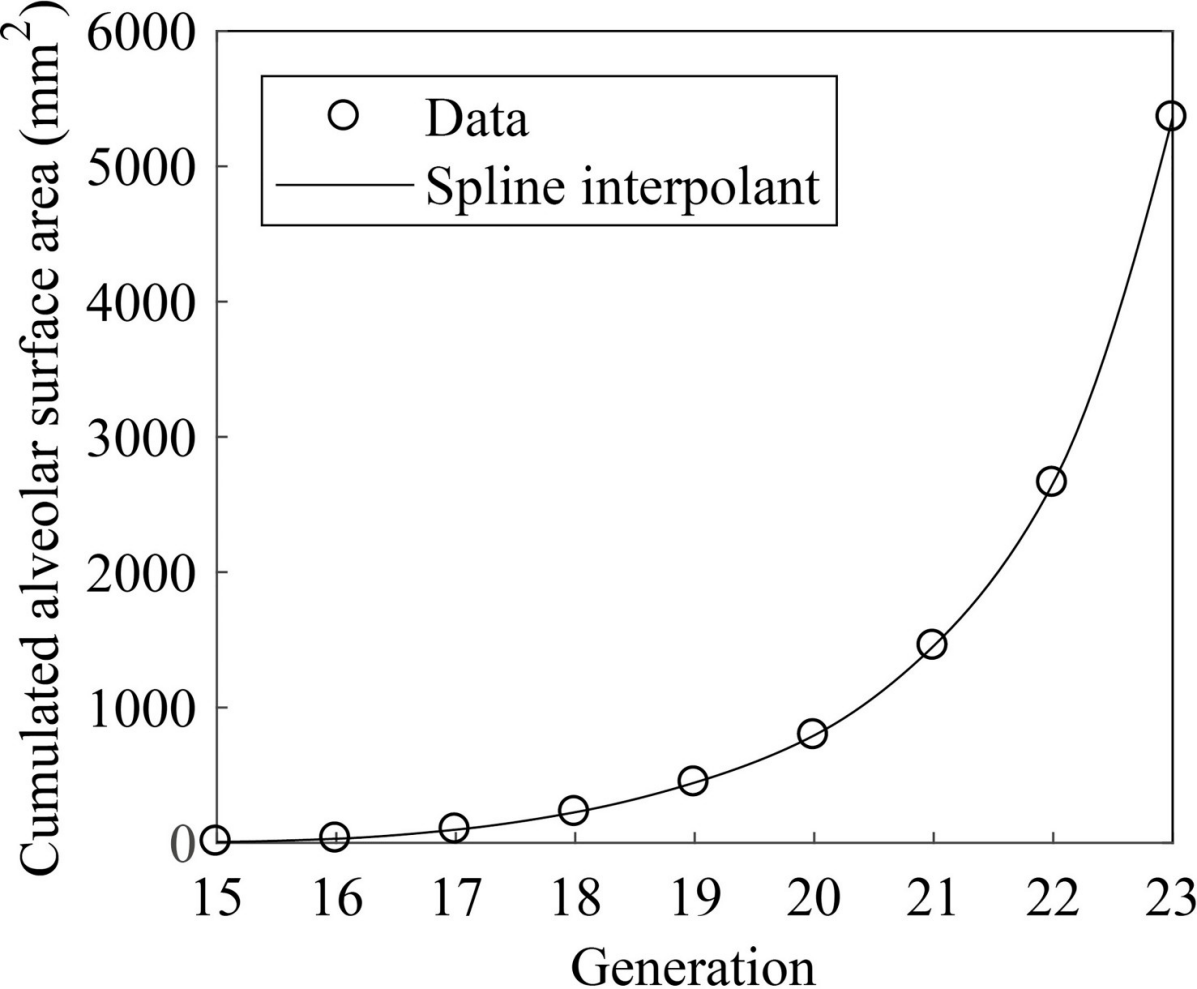
Cumulated alveolar surface area. The circles denote the data obtained from [12], and the line is a spline interpolant *A*_a_(*x*).

Alveoli mediate oxygen transfer from the airways to the capillaries. Alveoli begin to appear from the transitional bronchioles, but most alveoli are attached to distal airways. Fig 3 shows the cumulated alveolar surface area with respect to airway generation [14, 17]. Assuming that the airway length in each generation is 1 mm, we formulate the cumulated alveolar surface area as a continuous function *A*_a_(*x*) using spline interpolation, and the local alveolar surface area per unit length is given by *A*_a_′(*x*) = d*A*_a_/d*x*. One can calculate the local oxygen transfer rate through alveoli per length as $q^{\prime }(x)=\beta D_{w}(\varphi (x)-\varphi_{c})A_{a}^{\prime }(x)/h$, where *β* is the solubility of oxygen in water [17], *D*_w_ is the diffusivity of oxygen in air, *φ*(*x*) is the partial pressure of oxygen in the acinar airways, *φ*_c_ is the oxygen partial pressure in the blood capillary, and *h* is the thickness of the alveolar membrane.

We examine the oxygen conservation in an infinitesimal control volume, as shown in Fig 2. Because sidewalls of a single airway prevent diffusion to neighboring airways, we only consider longitudinal diffusion along the *x*-axis,

$$\frac{\partial \varphi }{\partial t}=D\left(\frac{\partial^{2}\varphi }{\partial x^{2}}+\frac{1}{a}\frac{\partial \varphi }{\partial x}\right)-\beta D_{w}\left(\frac{\varphi -\varphi_{c}}{h}\right)\left(\frac{A_{a}^{\prime }(x)}{A_{16}e^{x/a}}\right)$$ (1)

where *t* is the time after inhalation begins. Solving Eq (1) requires an initial condition and two boundary conditions. We assume that the oxygen partial pressure in the acinar airways is the same as that of the blood capillary when inhalation begins because an exhalation time of ~ 2 s is greater than the diffusion time scale *L*^2^/(4*D*) ~ 0.8 s, where *L* ~ 8 mm is the diffusion length from the 16th to 23rd airways. Thus, the initial condition is written as *φ*(*x*, 0) = *φ*_c_. Once inhalation begins, fresh air is supplied from the conducting airways. Accordingly, we assume that the oxygen partial pressure at the inlet of the 16th airway branch remains the same as that in the fresh air during the inhalation. This leads us to write a boundary condition as *φ*(0,*t*) = *φ*_a_, where *φ*_a_ is the oxygen partial pressure in the fresh air. The other boundary condition comes from the 23^rd^ generation where oxygen transfer is allowed only through the alveolar membrane, so that ${\left(\frac{\partial \varphi }{\partial x}\right)}_{x=L}=0$.

**Fig 4:**
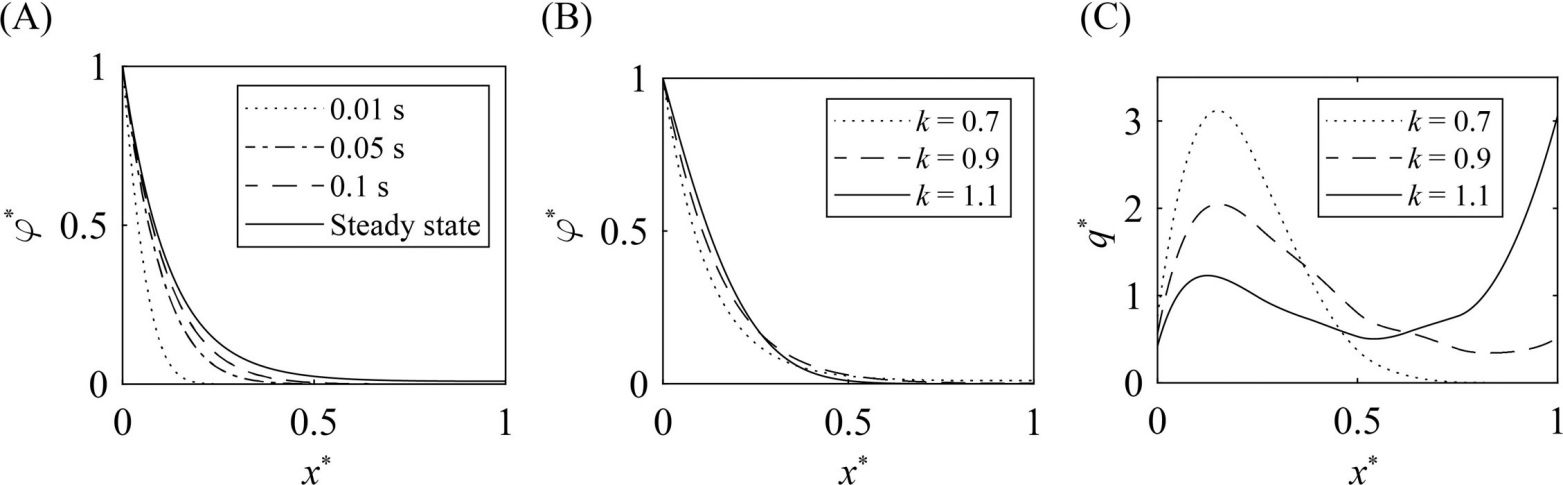
Profiles of oxygen partial pressure and oxygen transfer rate. (A) Temporal change in the distribution of the oxygen partial pressure for *k* = 0.9. (B) Profiles of the oxygen partial pressure for various diameter reduction ratios (*k* = 0.7, 0.9, and 1.1). (C) The dependence of the oxygen transfer to the blood capillaries on the airway depth. Note that the area below the curves is equal to unity by the definition of $\hat{q}$.

By numerically solving Eq (1), we obtain the profile of oxygen partial pressure in acinar airways during the inhalation (see Materials and methods). Fig 4A shows the dimensionless oxygen partial pressure $\hat{\varphi }=(\varphi -\varphi_{c})/(\varphi_{a}-\varphi_{c})$ as a function of $\hat{x}=x/L$ for *k* = 0.9, which approaches the steady state profile within ~0.1 s. Hence, we neglect the unsteady effects in estimating the oxygen transfer during the whole inhalation process. Fig 4B and 4C show the profiles of the partial pressure and normalized oxygen transfer rate $\hat{q}$ to the blood capillaries, respectively, for various diameter reduction ratios (*k* = 0.7, 0.9 and, 1.1). Here $\hat{q}$ is defined as

$$\hat{q}(\hat{x})=\frac{\left[\varphi (\hat{x})-\varphi_{c})]{\hat{A}}_{a}^{\prime }(\hat{x}\right)}{\int_{0}^{1}[\varphi (\hat{x})-\varphi_{c}]{\hat{A}}^{\prime }(\hat{x})d\hat{x}},$$ (2)

with ${\hat{A}}_{a}^{\prime }=(dA_{a}/d\hat{x})/A_{a}(\hat{x}=1)$ so that the total area below a curve $\hat{q}$ is equal to unity.

Acinar airways comprise a thin layer of epithelial cells covering airways and alveoli. Therefore, building an additional airway demands costs for construction and maintenance of the epithelial cell layer, which are proportional to the total surface area of the acinar airways. This reminds us of the fact that the expansion of the cross-sectional area in plant xylem vessels is limited by the carbon investment for the construction of the vessels [6]. We thus suggest that the total surface area of the acinar airways is the primary limiting factor.

**Fig 5:**
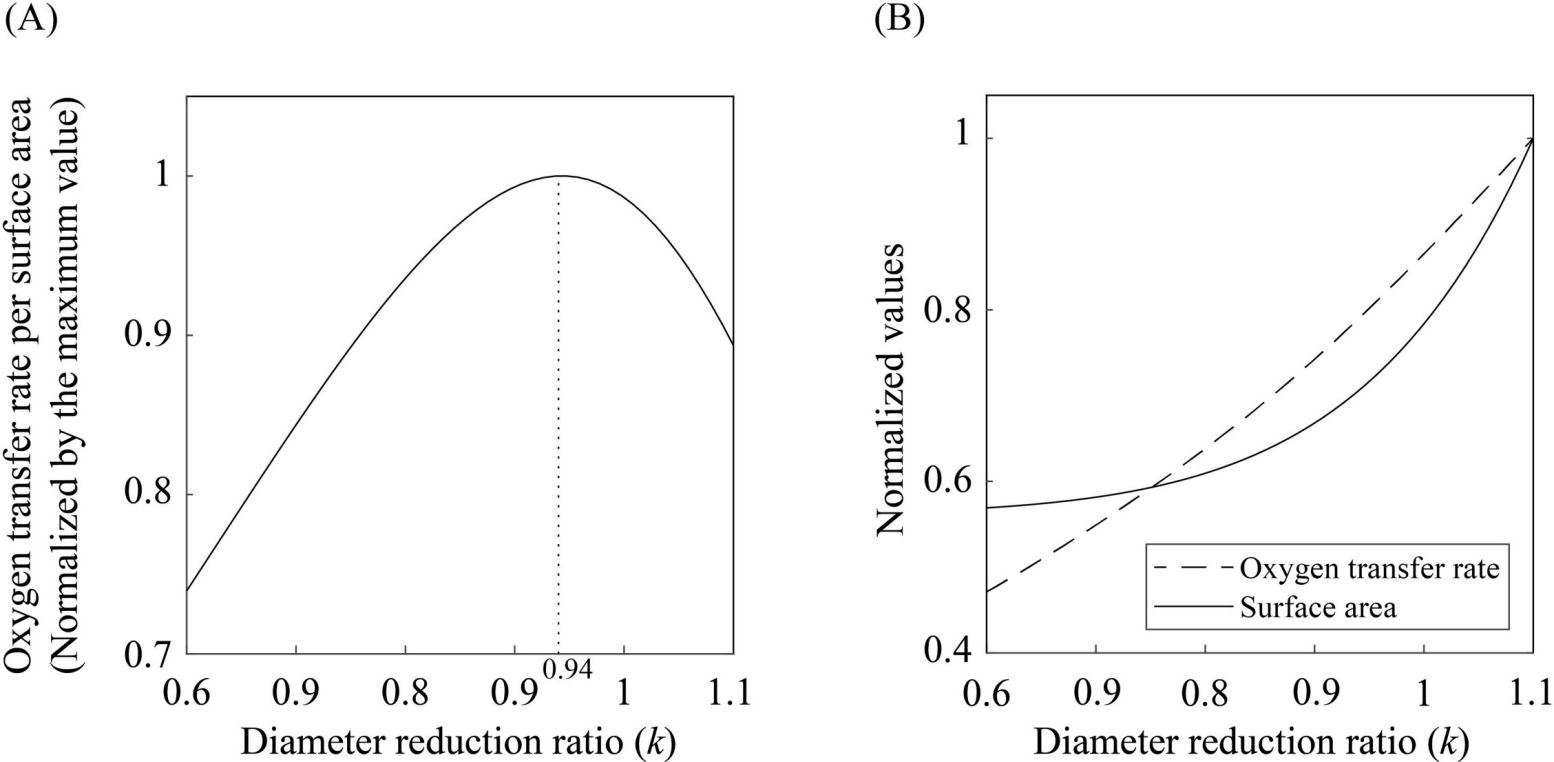
Optimal diameter reduction ratio for maximizing the oxygen transfer rate per airway surface area. (A) Oxygen transfer rate per surface area of acinar airways and alveoli versus diameter reduction ratio. The oxygen transfer rate per surface area peaks at a diameter reduction ratio of 0.94, for which the energy cost for transporting a given amount of oxygen is minimized, provided that the energy investment is proportional to the surface area of acinar airways. (B) The dependence of oxygen transfer rate and surface area of acinar airways on the diameter reduction ratio. The values are normalized by their maximum values, respectively.

Fig 5A displays the total oxygen transfer rate through the entire alveoli divided by the surface area of the acinar airways and alveoli versus the diameter reduction ratio. We see that although the total oxygen transfer rate increases with the diameter reduction ratio *k*, the sharp increase of the surface area with *k* results in the maximum *Q*/*A* at *k* = 0.94 (see Fig 5B). We thus infer that a ratio of 0.94 is the optimal reduction ratio to minimize the cost of the acinar airways for oxygen transfer. This theoretical value is indeed consistent with the biological data shown in Fig 1B. On the basis of the model, it can be deduced that the transition of the diameter reduction ratios between the conducting (*k* = 0.79) and acinar (*k* = 0.94) airways saves approximately 10% of the epithelial cell layers compared with the case without this transition. Consequently, our model suggests that this transition occurs for reducing energy and materials consumption in the acinar airways.

## Discussion

**Fig 6:**
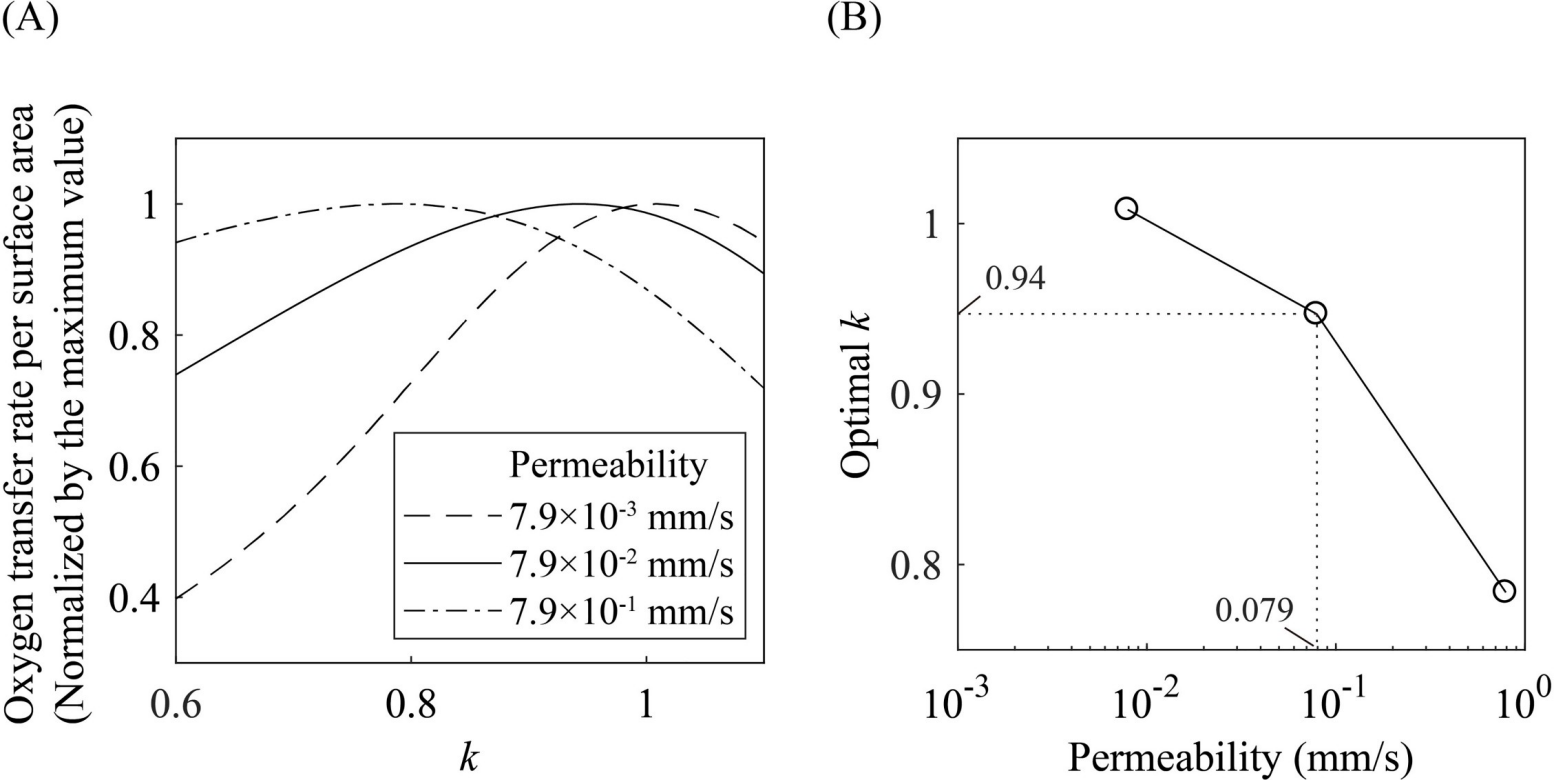
Dependence of optimal diameter reduction ratio on the alveolar membrane permeability. (A) Oxygen transfer rate per surface area versus diameter reduction ratio for various permeability. (B) The optimal diameter reduction ratios for various permeability.

The oxygen transfer to blood in acinar airways depends on the permeability of the alveolar membrane *βD*_w_/*h*, so that membrane permeability can change the optimal diameter reduction ratio. One can infer that the higher the permeability of the membrane, the less the surface required for a given amount of oxygen transfer. Hence, the more permeable alveoli would reduce the transfer through distal airways, referred to as ‘screening effects’ [17, 27], and reduce the effectiveness of distal airways. In Fig 6, we present the dependence of the oxygen transfer rate per surface area on the diameter reduction ratio for various permeability of the alveolar membrane. Indeed, the optimal diameter reduction ratio decreases with the membrane permeability, resulting in a smaller area of the terminal airways for higher permeability.

Originally developed for blood vessels, Murray’s law describes branching structure for minimizing energy cost for convective transport and metabolism of blood [9, 10]. This design principle of branching structure has been used as a framework to understand various natural network systems under the assumption that natural branching networks have evolved in such a way as to minimize the cost for transferring a given quantity of matter [6, 7]. Murray’s law for conducting airways of lung explains the minimization of energy consumption for oxygen transport for a given volume of air inhalation [8, 10, 17]. Murray’s law for plant xylem elucidates the minimization of the primary energetic cost caused by sap flow and vessel construction [11]. Interestingly, despite various forms of energetic costs, Murray’s law for these networks commonly consider convection transport energy and vessel volume. Murray’s law for diffusion on the other hand can be formulated mathematically by replacing the energy cost for convective transport with the energy cost for diffusive transport, leading to an optimal diameter reduction ratio of 0.71 [23, 24].

However, the biological data of acinar airways in human lungs cannot be explained by either of the aforementioned models. We here raise in this report the question of transition of diameter reduction effects on lung optimization. Specifically, we consider the amount of diffusive transport of oxygen per surface area of airways. This implies that the dominant cost for the construction and maintenance of acinar airways comes from epithelial cell layers of airways and alveoli, which should be measured by the surface area rather than the volume.

Although advection is typically cost effective on the organismal scale, diffusion is a rapid, reliable, and cheap way to transfer matter on the cell scale [1, 28]. In mammals relying on air breathing, the characteristic diameter of the terminal branches of the airways is on the order of 100 μm, regardless of the body size [14, 29, 30]. Thus, gas transport via diffusion would be more effective near the terminal branches. Accordingly, the transition of the oxygen transfer mode is also expected in the lung airways of other species, which may cause the transition of the diameter reduction ratios. Indeed, some anatomical data on the lung airways of other species, including rats, rabbits, and canines, show a transition in the diameter reduction ratio as in human lung airways [29, 30], suggesting an interesting aspect worth pursuing in the future. This optimal strategy of acinar airways can guide design and construction of artificial networks where one needs to maximize fluid transport to a given area [31–33].

## Materials and methods

We explain the process to solve Eq (1), the oxygen diffusion equation in the dichotomous hierarchical branch networks, subject to the boundary conditions:

$$\varphi (0,t)=\varphi_{a}\;\mathrm{and}\;{\frac{\partial \varphi }{\partial x}|}_{x=L}=0.$$ (3)

Using the following dimensionless variables

$$\hat{x}=\frac{x}{L},\;\hat{t}=\frac{Dt}{L^{2}},\;\hat{\varphi }=\frac{\varphi -\varphi_{c}}{\varphi_{a}-\varphi_{c}},\;\alpha_{1}=\frac{L}{a},\;\mathrm{and}\;\alpha_{2}(\hat{x})=\frac{\beta D_{w}L}{Dh}\frac{1}{A_{16}e^{(L/l)\hat{x}}}{\hat{A}}_{a}^{\prime }(\hat{x}),$$

we find dimensionless forms of Eqs (1) and (3):

$$\frac{\partial \hat{\varphi }}{\partial \hat{t}}=\frac{\partial^{2}\hat{\varphi }}{\partial {\hat{x}}^{2}}+\alpha_{1}\frac{\partial \hat{\varphi }}{\partial \hat{x}}-\alpha_{2}(\hat{x})\hat{\varphi }$$ (4)

$$\hat{\varphi }(\hat{x},0)=0,\;\hat{\varphi }(0,\hat{t})=1\;\mathrm{and}\;{\frac{\partial \hat{\varphi }}{\partial \hat{x}}|}_{\hat{x}=1}=0$$ (5)

Eq (4) with the initial and boundary conditions was solved numerically with parameters given in Table 1. Spatial derivatives in Eq (4) was discretized on uniformly spaced spatial grids (*N* = 100) using the second-order central difference scheme to obtain a system of ordinary differential equations (ODE) corresponding to a type of initial value problem. The resulting system of ODE is solved by a multistep method.

**Table 1:** Geometrical data of acinar airways [12, 17].

Variable	Definition	Unit	Value
*l*	Length of each airway branch	mm	1
*A*_16_	Surface area of first generation of acinar airways	mm^2^	4.18
*h*	Capillary membrane thickness	mm	0.001
*D*	Diffusion coefficient of oxygen in air	mm^2^/s	20
*D*_w_	Diffusion coefficient of oxygen in water	mm^2^/s	0.0033
*β*	Solubility of oxygen in water	1	0.024
